# Investigating the Effectiveness of Spatial Frequencies to the Left and Right of Central Vision during Reading: Evidence from Reading Times and Eye Movements

**DOI:** 10.3389/fpsyg.2017.00807

**Published:** 2017-07-18

**Authors:** Timothy R. Jordan, Victoria A. McGowan, Stoyan Kurtev, Kevin B. Paterson

**Affiliations:** ^1^Department of Psychology, Zayed University Dubai, United Arab Emirates; ^2^Department of Neuroscience, Psychology, and Behaviour, University of Leicester Leicester, United Kingdom; ^3^Centre for Research in Psychology, Behaviour and Achievement, Coventry University Coventry, United Kingdom

**Keywords:** reading, eye movements, perception, language, cognition

## Abstract

Printed words are complex visual stimuli containing a range of different spatial frequencies, and several studies have suggested that various spatial frequencies are effective for skilled adult reading. But while it is well known that the area of text from which information is acquired during reading extends to the left and right of each fixation, the effectiveness of spatial frequencies falling each side of fixation has yet to be determined. To investigate this issue, we used a spatial frequency adaptation of the gaze-contingent moving-window paradigm in which sentences were shown to skilled adult readers either entirely as normal or filtered to contain only low, medium, or high spatial frequencies except for a window of normal text around each point of fixation. Windows replaced filtered text either symmetrically 1 character to the left and right of each fixated character, or asymmetrically, 1 character to the left and 7 or 13 to the right, or 1 character to the right and 7 or 13 to the left. Reading times and eye-movement measures showed that reading performance for sentences presented entirely as normal generally changed very little with filtered displays when windows extended to the right but was often disrupted when windows extended to the left. However, asymmetrical windows affected performance on both sides of fixation. Indeed, increasing the leftward extent of windows from 7 to 13 characters produced decreases in both reading times and fixation durations, suggesting that reading was influenced by the spatial frequency content of leftward areas of text some considerable distance from fixation. Overall, the findings show that while a range of different spatial frequencies can be used by skilled adult readers, the effectiveness of spatial frequencies differs for text on each side of central vision, and may reflect different roles played by these two areas of text during reading.

## Introduction

Fluent reading relies on making saccadic eye movements and ending each movement with a brief fixational pause during which time visual information is acquired from the text (for reviews, see Rayner, 1998, 2009). However, the precise nature of this visual information, and the area of text within which this information affects reading (usually referred to as the *perceptual span*), have yet to be fully revealed.

Of particular importance for understanding the influence of the perceptual span is that a great deal of evidence indicates that human vision operates in the spatial frequency domain, and neural pathways exist that show selective sensitivity to spatial frequencies associated with different scales of visual information (e.g., Robson, 1966; Blakemore and Campbell, 1969; Lovegrove et al., 1980). As a result, when reading, the visual system acquires a range of spatial frequencies from text during each fixational pause, and these spatial frequencies provide the bases for the subsequent linguistic analyses that ultimately allow readers to obtain meaning from what they are seeing (e.g., Lovegrove et al., 1980; Patching and Jordan, 2005a,b; Allen et al., 2009; Jordan et al., 2016a,b). For example, lower spatial frequencies allow readers to see a word’s overall shape but not its fine detail, whereas higher spatial frequencies allow readers to see fine detail, such as the precise form of letter strokes, but are less useful for seeing a word’s overall shape (e.g., Legge et al., 1985; Jordan, 1990, 1995; Patching and Jordan, 2005a,b; Allen et al., 2009; Kwon and Legge, 2012; Jordan et al., 2016a,b). Thus, although spatial frequency analyses are not apparent to the reader, reading relies fundamentally on these low-level visual properties of text.

But the effectiveness of spatial frequencies for reading and, in particular, how this effectiveness changes around the point of gaze, have yet to be fully established. In particular, since the pioneering studies of Keith Rayner and his colleagues (e.g., McConkie and Rayner, 1975, 1976; Rayner et al., 1980, 1982; Underwood and McConkie, 1985), estimates of the perceptual span have been obtained using a gaze-contingent moving-window paradigm in which text extending leftward or rightward from each point of fixation is displayed normally during reading while text lying beyond these areas is replaced (usually by substituting different letters). Using this technique, numerous studies have proposed that the perceptual span for skilled reading of English, and other alphabetic systems read from left to right, is asymmetrical, and extends around 14 characters to the right of fixation but no more than 3–4 characters to the left, and certainly no further than the beginning of the fixated word (e.g., McConkie and Rayner, 1975, 1976; Rayner et al., 1980, 1982; Underwood and McConkie, 1985). More recently, however, further investigations suggest that the leftward span is larger than previously believed (e.g., Binder et al., 1999; Rayner et al., 2009; Apel et al., 2012; Jordan et al., 2013, 2016), and may actually extend at least 12 characters to the left of fixation (Jordan et al., 2013, 2016). Consequently, it is now sensible to consider that the extent of the perceptual span may be more broadly symmetrical to the left and right of fixation, and that information from both these areas contributes to normal reading performance.

Yet the effectiveness of the spatial frequencies present in text to the left and right of fixation during reading is largely unknown. What we do know from several recent investigations (Jordan et al., 2012, 2014b, 2016a,b; Paterson et al., 2012, 2013a,b) is that when entire lines of text are filtered so that only certain spatial frequencies remain, readers use a broad range of different spatial frequencies. Indeed, reading performance for young skilled adult readers is often close to normal when lines of text contain only medium to very high spatial frequencies but reading still occurs even when only low spatial frequencies are present and so detailed information is absent. However, the purpose of these previous studies was to assess the influence of spatial frequencies on reading performance by using entire lines of filtered text, and the effectiveness of spatial frequencies either side of fixation remains to be explored.

There is good reason to expect that the effects on reading exerted by the spatial frequency content of text differs for spatial frequencies encoded from the left and right of fixation. In particular, a major requirement for reading languages from left to right is to obtain visual information from words in areas of text to the right of fixation so that upcoming words can be readily identified and forward progression can take place effectively and efficiently. Consequently, as part of this process, reading requires visual input from text to the right of fixation that can help determine accurately the words that are encountered in these locations (e.g., Rayner, 2009). In contrast, when these words are subsequently passed by a forward saccade, which places them to the left of the new fixation location, the visual content of these words is likely to have been processed sufficiently for them to be identified (e.g., Reichle et al., 2006) and so the spatial frequencies present in leftward areas of text may play a different role in reading. Indeed, several researchers have argued that a major requirement of processing leftward text is to maintain a record of the identities and locations of words in these locations which helps preserve the linguistic and spatial content of each line of text as it is read (see Kennedy et al., 2003; Mitchell et al., 2008; Jordan et al., 2013, 2016). Consequently, whereas processing text to the right of fixation requires sufficient spatial frequency input to help determine precisely the identities of novel words, the role of spatial frequency input from text to the left of fixation may be less demanding, requiring only sufficient cues to help monitor words that have already been identified.

The effectiveness of the spatial frequency content of text to the left and right of fixation is also likely to be influenced, to some extent, by asymmetries in the processing abilities of the two cerebral hemispheres. In particular, because of the anatomical arrangement of human vision, large areas of text falling to the left and right of each fixation when reading will project unilaterally to each contralateral hemisphere (for reviews, see Gazzaniga, 2000; Jordan and Paterson, 2009, 2010). So although a small area of overlap exists around each fixation within which text projects bilaterally to both hemispheres (see e.g., Gazzaniga, 2000; Jordan and Paterson, 2009, 2010; Jordan et al., 2010a,b, 2011b; Almabruk et al., 2011), rightward text and leftward text outside this area of central vision will project unilaterally to the left or right hemispheres, respectively.^1^ One consequence of this arrangement is that text to the right of central vision will project to the left hemisphere, which is generally dominant for processing language (e.g., Knecht et al., 2000; Denes, 2016), and numerous studies using lateralized displays suggest that this produces a processing advantage for words encountered to the right of fixation (for reviews, see Jordan et al., 1998, 2000, 2003; Gazzaniga, 2000). But many studies also show that the left hemisphere processes high spatial frequencies more quickly and more accurately than the right hemisphere, whereas the right hemisphere processes low spatial frequencies more quickly and more accurately than the left (Christman et al., 1991; Hellige, 1993; Christman, 1997; Peyrin et al., 2003; see also Martínez et al., 2001; Musel et al., 2013; Piazza and Silver, 2014). As a result, not only is it likely that processing leftward and rightward text when reading is affected by differential access to the language-dominant left hemisphere, it may also be the case that processing leftward and rightward text is affected differently by the spatial frequency content present each side of central vision.

**FIGURE 1:**
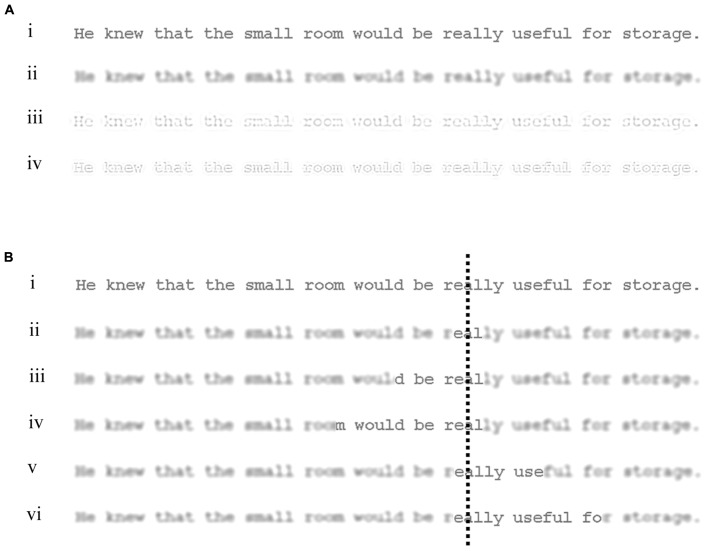
Examples of the types of display used in the experiment. **(A)** Shows a sentence displayed as (i) normal, and filtered to contain only (ii) low, (iii) medium, or (iv) high spatial frequencies. **(B)** Illustrates the normal and 5 window conditions used in the experiment: (i) Normal, (ii) L1-R1, (iii) L7-R1, (iv) L13-R1, (v) L1-R7, and (vi) L1-R13. For this illustration, a low-spatial frequency display has been used. The dashed line represents an example fixation location and was not shown in the actual experiment. Note that the visual appearance of the filtered text in the figure is approximate due to restrictions in resolution and print medium.

Accordingly, the purpose of the present research was to help determine the effectiveness of the spatial frequency content of text to the left and right of central vision when reading sentences that were filtered to contain just one band of spatial frequencies. To do this, we used a spatial-frequency variation of McConkie and Rayner’s (1976) gaze-contingent moving-window paradigm in which sentences were filtered to contain only low, medium, or high spatial frequencies except for a window of normal text that extended to the left and right of each fixated character (see **Figure 1**). The location of the window was yoked to the reader’s gaze so that, as the eyes moved to a new location, the window moved in synchrony with these movements, and was displayed at the new gaze location while all text outside the new window contained only the spatial frequency band used for that display. In this way, the area within which filtered text was replaced by normal text during reading could be controlled while preserving the word lengths, word boundaries, and identities of words and letters present in each sentence.

Across window conditions, the width of the window (and so the amount of filtered text that was replaced by normal text) was increased outward from the point of fixation (**Figure 1**). The smallest window condition contained a small area of normal text which extended symmetrically in central vision around each fixated character. This condition provided a baseline against which effects of extending windows asymmetrically to the left or right of fixation could be compared. In the asymmetrical window conditions, the area of normal text was increased to either 7 or 13 characters to the right, or 7 or 13 to the left. Using these conditions, the areas of filtered text replaced each side of the point of gaze could be manipulated to reveal the relative effectiveness of each type of spatial frequency in these areas when reading.

The logic of this approach to understanding the role of spatial frequencies in reading is straightforward. If the spatial frequency band present in a filtered sentence display each side of central vision is sufficient for normal reading, replacing this filtered text with normal text should produce no change in reading performance. However, if the spatial frequency band present each side of central vision is not sufficient for normal reading, replacing this filtered text with normal text should show improvements in reading performance. Moreover, if the effectiveness of spatial frequencies for reading differs to the left and right of central vision, this difference should be revealed by different effects produced when filtered text is replaced by normal text on each side of fixation. In particular, if certain spatial frequencies (e.g., low) are less effective to the right of central vision than to the left, replacing these spatial frequencies with normal text should show the greatest improvement in reading performance when these replacements are to the right of central vision rather than to the left. Indeed, from the arguments we have already made, processing text to the right of fixation is likely to require sufficient spatial frequency input to help determine precisely the identities of novel words, whereas the role of spatial frequency input from text to the left of fixation may be less demanding, requiring only sufficient cues to help monitor words that have already been identified.

## Materials and Methods

### Ethics Statement

This study was conducted in accordance with the recommendations of the Research Ethics Committee of the University of Leicester, with written informed consent from all participants, in accordance with the Declaration of Helsinki. The protocol was approved by the Research Ethics Committee of the University of Leicester.

### Participants

Sixteen participants (aged 18–30 years) were recruited from the University of Leicester and local community. All participants were native speakers of English and had normal or corrected-to-normal vision, as determined by Bailey-Lovie (Bailey and Lovie, 1980), ETDRS (Ferris and Bailey, 1996), and Pelli-Robson (Pelli et al., 1988) assessments (see Jordan et al., 2011c).

### Stimuli and Design

One hundred and sixty sentences were displayed either entirely as normal or filtered using MATLAB to leave just one of three different, 1-octave wide bands of spatial frequencies in each sentence display, with center (peak) frequencies of 3.5, 6.7, and 11.1 cycles per degree (cpd); these were termed *low, medium*, and *high* spatial frequencies, respectively (see Patching and Jordan, 2005a,b). These three bands of spatial frequencies are known to be influential in word recognition and textual reading (e.g., Patching and Jordan, 2005a,b; Jordan et al., 2012, 2014b, 2016a,b; Paterson et al., 2012, 2013a,b) and so were well-suited to revealing differences in the use of the spatial frequency content of text each side of fixation. Sentences were 49–65 characters in length, of various structures, and did not include any syntactically anomalous items.

For each filtered display, a window of normal text was shown yoked to the reader’s gaze so that when the eyes moved to a new fixation location, the window moved in synchrony with these movements and a new window of normal text was displayed at the new gaze location while all text outside this new window was presented in the spatial frequency band (low, medium, or high) used for that display. Five types of window were used (see **Figure 1**). In the smallest window condition (L1-R1), normal text extended symmetrically one character to the left and right of each fixated character. In the asymmetrical window conditions, normal text extended 1 character to the left and either 7 (L1-R7) or 13 (L1-R13) to the right, or 1 character to the right and either 7 (L7-R1) or 13 (L13-R1) to the left.

### Apparatus and Procedure

Eye movements were recorded using an Eyelink 2K tower-mounted eye-tracker with chin and forehead rest. Viewing was binocular and each participant’s right-eye movements were sampled at 1000 Hz using pupil tracking and corneal reflection. Sentences were displayed on a high-definition 19-inch monitor with a screen refresh rate of 120 Hz and a 4-letter word subtended approximately 1° (i.e., normal size for reading; Rayner and Pollatsek, 1989). The eye-tracker was calibrated at the beginning of the experiment and calibration was checked between trials and the tracker was recalibrated as necessary. At the start of each trial, a fixation square equal in size to one character was presented on the left of the screen. Once the participant fixated this location accurately for 250 ms, a sentence was presented with its first letter replacing the fixation square. Participants were instructed to read normally and for comprehension and pressed a response key as soon as they finished reading each sentence. The sentence was then replaced by a comprehension question, to which participants responded. To provide a comprehensive measure of the influence of different areas of text on reading, reading performance was assessed by recording overall sentence reading time (measured from the onset of a sentence display to the response key press), mean fixation durations (the average length of fixational pauses), total number of fixations (the number of these fixational pauses), regressive saccade count (the number of backward movements in the text), and the length of progressive saccades. These measures are standard in eye-movements research (see Rayner, 2009).

## Results

**FIGURE 2:**
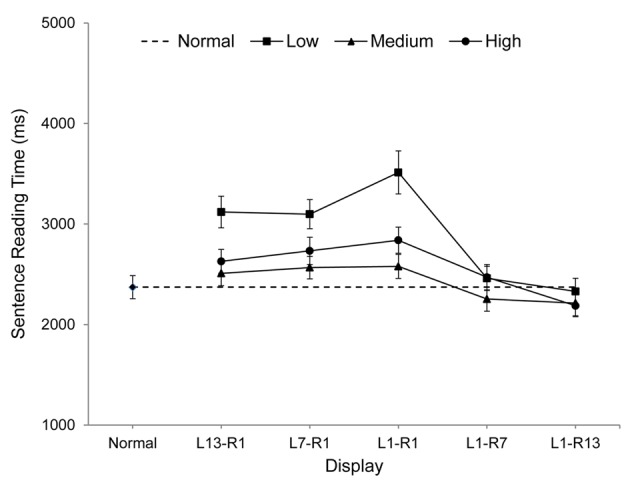
Mean Sentence Reading Times (including standard error bars) for each display condition.

All participants scored 80% or better on the comprehension questions (mean = 91%), indicating that sentences were read normally. Sentence reading times provide the most complete and informative measure of overall reading performance in eye movement experiments and these are shown graphically in **Figure 2**. Sentence reading times, fixation durations, number of fixations, number of regressions, and progressive saccade length are also reported in **Table 1**. For each measure, the data for each spatial frequency band (low, medium, high), were analyzed using Analyses of Variance to compare performances for the five window conditions and the normal text condition, with error computed across participants (*F*_1_).^2^

**Table 1:** Mean reading times and eye movement measures for each display condition.

	Normal	Spatial Frequency	L13-R1	L7-R1	L1-R1	L1-R7	L1-R13
Reading Time	2372 (115)	Low	3120 (157)	3098 (145)	3513 (214)	2460 (120)	2330 (131)
		Medium	2510 (123)	2567 (112)	2579 (120)	2254 (121)	2214 (126)
		High	2629 (119)	2733 (136)	2839 (130)	2471 (126)	2187 (108)
Fixation Duration	228 (6)	Low	262 (7)	265 (6)	285 (8)	243 (6)	239 (5)
		Medium	236 (6)	239 (7)	248 (6)	233 (6)	233 (5)
		High	244 (5)	244 (5)	255 (6)	234 (5)	228 (5)
Fixation Count	8.9 (0.4)	Low	10.5 (0.5)	10.4 (0.5)	10.9 (0.6)	8.9 (0.5)	8.4 (0.4)
		Medium	9.3 (0.5)	9.4 (0.4)	9.1 (0.4)	8.4 (0.4)	8.2 (0.4)
		High	9.4 (0.4)	9.7 (0.4)	9.7 (0.4)	9.1 (0.4)	8.2 (0.4)
Regressions	2.1 (0.3)	Low	2.1 (0.2)	2.0 (0.2)	2.2 (0.2)	1.8 (0.2)	1.8 (0.2)
		Medium	1.6 (0.2)	1.8 (0.2)	1.6 (0.2)	1.4 (0.2)	1.7 (0.2)
		High	1.4 (0.2)	1.7 (0.2)	1.6 (0.3)	1.7 (0.2)	1.7 (0.2)
Progressive Saccade Amplitude	10.3 (0.4)	Low	8.7 (0.4)	8.7 (0.4)	8.4 (0.4)	9.4 (0.4)	10.4 (0.4)
		Medium	9.0 (0.4)	9.3 (0.4)	9.1 (0.4)	9.6 (0.4)	10.5 (0.4)
		High	8.4 (0.4)	8.6 (0.4)	8.6 (0.4)	9.1 (0.4)	10.2 (0.4)

*Saccade amplitudes are shown in characters and Standard Errors are in parentheses.*

### Reading Times

An overall interaction was observed between window condition and spatial frequency band, *F*_1_(8,240) = 10.61, *p* < 0.001, $\eta_{p}^{2}$ = 0.26, and further analyses showed an effect of window condition for each spatial frequency [low, *F*_1_(5,75) = 56.91, *p* < 0.001, $\eta_{p}^{2}$ = 0.79; medium, *F*_1_(5,75) = 11.19, *p* < 0.001, $\eta_{p}^{2}$ = 0.43; high, *F*_1_(5,75) = 16.46, *p* < 0.001, $\eta_{p}^{2}$ = 0.52].

For low spatial frequencies, reading times within the five window conditions were longest for L1-R1, equally shorter for L13-R1 and L7-R1, shorter still for L1-R7, and shortest of all for L1-R13 (all *p*s < 0.05). In addition, reading times for L1-R7 and L1-R13 did not differ from those obtained for normal displays (*p*s > 0.29) but were longer than normal for all other windows (*p*s < 0.01).

For medium spatial frequencies, reading times within window conditions were longest for L1-R1, L7-R1 and L13-R1, and equally shortest for L1-R7 and L1-R13 (*p*s < 0.05). Reading times also did not differ from normal for L1-R7, L1-R13, and L13-R1 (all *p*s > 0.13) but were longer than normal for L7-R1 and L1-R1 (*p*s < 0.01).

For high spatial frequencies, reading times within window conditions were longest for L1-R1, equally shorter for L7-R1 and L13-R1, shorter still for L1-R7, and shortest for L1-R13. Reading times were also shorter than normal for L1-R13 (*p* < 0.05), did not differ from normal for L1-R7 (*p* = 0.26), and were longer than normal for all other windows (*p*s < 0.05).

### Fixation Durations

An overall interaction was observed between window condition and spatial frequency band, *F*_1_(8,240) = 7.01, *p* < 0.001, $\eta_{p}^{2}$ = 0.19, and further analyses showed an effect of window condition for each spatial frequency [low, *F*_1_(5,75) = 40.35, *p* < 0.001, $\eta_{p}^{2}$ = 0.73; medium, *F*_1_(5,75) = 5.98, *p* < 0.001, $\eta_{p}^{2}$ = 0.29; high, *F*_1_(5,75) = 14.46, *p* < 0.001, $\eta_{p}^{2}$ = 0.49].

For low spatial frequencies, fixation durations within window conditions were longest for L1-R1, equally shorter for L7-R1 and L13-R1, and shortest of all for L1-R7 and L1-R13 (all *p*s < 0.01). In addition, fixation durations did not differ from normal for L1-R13 (*p* > 0.05) but were longer than normal for all other windows (*p*s < 0.01).

For medium spatial frequencies, fixation durations within window conditions were longest for L1-R1, and equally shorter for all other windows (*p*s < 0.05). Fixation durations did not differ from normal for L13-R1, L1-R7 and L1-R13 (all *p*s > 0.10) but were longer than normal for L7-R1 and L1-R1 (*p*s < 0.05).

For high spatial frequencies, fixation durations within window conditions were longest for L1-R1, equally shorter for L7-R1 and L13-R1, shorter still for L1-R7, and shortest of all for L1-R13 (all *p*s < 0.05). Fixation durations did not differ from normal for L1-R7 and L1-R13 (*p*s > 0.12) but were longer than normal for all other windows (*p*s < 0.01).

### Fixation Count

An overall interaction was observed between window condition and spatial frequency band, *F*_1_(8,240) = 7.99, *p* < 0.001, $\eta_{p}^{2}$ = 0.21, and further analyses showed an effect of window condition for each spatial frequency [low, *F*_1_(5,75) = 23.89, *p* < 0.001, $\eta_{p}^{2}$ = 0.61; medium, *F*_1_(5,75) = 8.16, *p* < 0.01, $\eta_{p}^{2}$ = 0.35; high, *F*_1_(5,75) = 8.31, *p* < 0.001, $\eta_{p}^{2}$ = 0.36].

For low spatial frequencies, fixation counts within window conditions were highest of all for L1-R1, equally lower for L7-R1 and L13-R1, lower still for L1-R7, and lowest of all for L1-R13 (*p*s < 0.05). Fixation counts did not differ from normal for L1-R7 and L1-R13 (*p* = 0.06), but were higher than normal for all other windows (*p*s < 0.01).

For medium spatial frequencies, fixation counts within window conditions were equally highest for L1-R1, L7-R1, and L13-R1, and equally lowest for L1-R7 and L1-R13 (*p*s < 0.05). Fixation counts did not differ from normal for any window conditions (all *p*s > 0.05).

For high spatial frequencies, fixation counts within window conditions were equally highest for L1-R1, L7-R1, and L13-R1, and equally lower for L1-R7 and L1-R13 (*p* < 0.05). Fixation counts were higher than normal for L1-R1 (*p* < 0.05), lower than normal for L1-R13 (*p* < 0.01), and did not differ significantly from normal for any other window conditions (*p*s > 0.05).

### Regressions

An overall interaction was observed between window condition and spatial frequency band, *F*_1_(8,240) = 9.48, *p* < 0.001, $\eta_{p}^{2}$ = 0.24, and further analyses showed an effect of window condition for each spatial frequency [low, *F*_1_(5,75) = 2.34, *p* < 0.05, $\eta_{p}^{2}$ = 0.14; medium, *F*_1_(5,75) = 4.55, *p* < 0.01, $\eta_{p}^{2}$ = 0.23; high, *F*_1_(5,75) = 4.81, *p* < 0.001, $\eta_{p}^{2}$ = 0.24].

For low spatial frequencies, regressions within window conditions were fewest for L1-R7 and L1-R13 and equally more for all other windows (*p*s < 0.01). Number of regressions did not differ from normal for any windows (*p* > 0.20).

For medium spatial frequencies, regressions within window conditions were fewest for L1-R7 and equally more for all other windows (*p*s < 0.01). All window conditions produced fewer regressions than normal displays (*p*s < 0.05).

For high spatial frequencies, regressions did not differ across window conditions (*p*s > 0.05) and all window conditions produced fewer regressions than normal displays (*p*s < 0.05).

### Progressive Saccade Length

An overall interaction was observed between window condition and spatial frequency band, *F*_1_(8,240) = 4.46, *p* < 0.001, $\eta_{p}^{2}$ = 0.13, and further analyses showed an effect of window condition for each spatial frequency [low, *F*_1_(5,75) = 25.18, *p* < 0.001, $\eta_{p}^{2}$ = 0.63; medium, *F*_1_(5,75) = 20.43, *p* < 0.001, $\eta_{p}^{2}$ = 0.58; high, *F*_1_(5,75) = 26.97, *p* < 0.001, $\eta_{p}^{2}$ = 0.64]. For low spatial frequencies, progressive saccades within window conditions were longest for L1-R13, shorter for L1-R7, and equally shortest for L13-R1, L7-R1, and L1-R1 (all *p*s < 0.01). Progressive saccades were shorter than normal for all windows (*p*s < 0.01) except for L1-R13, which was the same as normal. Similarly, for medium and high spatial frequencies, progressive saccades within window conditions were longest for L1-R13, shorter for L1-R7, and equally shortest for L13-R1, L7-R1 and L1-R1 (*p*s < 0.05). Progressive saccades were shorter than normal for all windows (*p*s < 0.01) except for L1-R13, which was the same as normal.

## Discussion

The results of this study reveal substantial asymmetries in the influence of spatial frequencies to the left and right of central vision during reading. Overall, when sentences contained only low, medium, or high spatial frequencies except for a small, three-character area of normal text (L1-R1) centered at the point of each fixation, performance was generally poorer than when sentences were presented entirely as normal. But the findings obtained using asymmetrical windows showed that when windows of normal text were extended to replace filtered areas further to the right of center, reading times were reduced substantially. Indeed, for all bands of spatial frequencies, not only did windows L1-R7 and L1-R13 produce the fastest reading times of all window conditions but both rightward windows produced reading times that were no slower than when sentences were presented entirely as normal. The indication from this is that, provided normal text extended at least seven characters to the right of fixation, low, medium, and high spatial frequencies in the remaining areas of each line of text were each sufficient to support normal reading performance.^3^ In contrast, replacing filtered areas to the left of fixation produced benefits that were more limited. In particular, while windows L13-R1 and L7-R1 each produced faster reading times relative to the baseline (L1-R1) window condition, reading times for these leftward window conditions were longer than those observed for rightward window conditions, and were generally longer than for normal sentence displays. It appears that while the contribution normally made to reading by rightward areas of text relies on more than a single band of spatial frequencies (and may even require the full complement normally present in text), the contribution made by leftward areas is less sensitive to the spatial frequency content of visual input.

These findings for reading times were complemented by other measures of reading behavior. For example, across all spatial frequencies, fixations were generally shorter and occurred less often for L1-R7 and L1-R13 than for all other window conditions, and both rightward windows were capable of producing fixation behavior for all spatial frequencies that was the same as when sentences were entirely normal. In contrast, leftward windows were generally more beneficial for fixation performance when medium and high spatial frequency displays were used. In particular, for medium spatial frequency displays, L7-R1 and L13-R1 windows produced fixation durations that were no different from those produced by each rightward window, and were no longer than normal when windows extended 13 characters to the left (L13-R1). In addition, when normal text extended either 7 or 13 characters to the left, medium and high spatial frequency displays each produced fixation counts that were no different from those observed for normal displays (although they were slightly greater than for rightward windows). This pattern suggests that areas of normal text extending up to 13 characters to either the left or right of fixation can each be highly effective for reading provided that medium or high spatial frequencies can be encoded from other areas along the same line. When low spatial frequency displays were presented, however, normal fixation behavior was apparent only when rightward windows were used, indicating the greater effectiveness of processing low spatial frequencies in text to the left of fixation than to the right.

The evidence from progressive saccades and regressions provides further indications of the asymmetrical contribution of spatial frequencies to reading. In particular, for all spatial frequencies, progressive saccades were shorter than normal for all window conditions except when normal text extended 13 characters to the right of fixation, which produced progressive saccade lengths that were no different from those observed for normal displays. This suggests that normal forward saccades when reading English are largely insensitive to spatial frequency content to the left of fixation (and elsewhere along a line of text), but require a region of normal text extending up to 13 characters to the right. This is consistent with the view that a major requirement for reading languages from left to right is to obtain sufficient visual information from text to the right of fixation so that upcoming words can be identified readily, and forward progression of the eyes can take place effectively and efficiently (e.g., Rayner, 2009). The evidence from the present findings is that this process requires a richness of input from text to the right of fixation (encompassing roughly the fixated word and the word to its right) that is greater than a single band of spatial frequencies. In contrast, provided this rightward input is available, it seems that the natural content of low, medium, and high spatial frequencies in text to the left of fixation (and elsewhere) can each fulfill the requirements for normal forward saccadic progression. Thus, and in line with the other findings we have reported, while forward saccades rely critically on rich visual input from text to the right of fixation, the requirements of text elsewhere are far less critical.

But an important qualification of this view is that regressions (i.e., leftward-moving saccades) benefited particularly from the presence of low spatial frequency displays. Indeed, regression rates were normal for all windows in low spatial frequency displays but no normal regression rates were produced by any windows in medium or high spatial frequency displays. Previous researchers have argued that text to the left of fixation plays an important role in reading by maintaining information about the location of words that have already been read (e.g., see Kennedy et al., 2003; Mitchell et al., 2008; Jordan et al., 2016), and the current findings suggest that low spatial frequencies may provide this information well whereas medium and high spatial frequencies are less able to do so. This finding, in particular, is consistent with the view that low spatial frequencies to the left of fixation may be processed especially well due to their projection to the right hemisphere (see Introduction). But aspects of visual acuity are also likely to play a part, since low spatial frequencies are visible at some considerable distance from fixation whereas constraints imposed by the visual system render high and medium spatial frequencies much less visible as eccentricities increase (see Jordan and Paterson, 2009).

The findings of this study complement recent investigations of the role of spatial frequencies in reading (Jordan et al., 2012, 2014b, 2016a,b; Paterson et al., 2012, 2013a,b) and extend this work by indicating that while a range of spatial frequencies can be used by skilled adult readers, the effectiveness of these spatial frequencies for supporting component processes in reading differs each side of central vision. Indeed, the effects of asymmetrical windows were often more subtle to the left than to the right but it would be misguided to regard this difference as a lack of involvement of leftward areas of text during reading. In particular, previous findings indicate that the leftward area from which information affects reading extends at least 12 characters from fixation (Jordan et al., 2013, 2016; see also Binder et al., 1999; Rayner et al., 2009; Apel et al., 2012) and findings from the current study also indicate that influences on reading performance extend further leftward than the traditional notion of just 3–4 characters. In fact, increasing the leftward extent of windows from 7 to 13 characters produced decreases in both reading times and fixation durations when medium spatial frequency displays (but not low or high spatial frequency displays) were presented. This suggests that areas of text to the left were influential more than seven characters (and perhaps up to 13 characters) from fixation during reading and that these influences were sensitive to the spatial frequency content of these areas. But although influential, it seems unlikely that leftward areas of text are used for reading in the same way as text to the right. As we have already discussed, a major requirement of forward-directed processes that drive the eyes along a line of text is to obtain new information about words that have yet to be identified. Consequently, forward-directed attention and previews of upcoming text are important components of the rightward extent of the perceptual span (e.g., Rayner, 2009). In contrast, the requirements for reading placed on information from text to the left of fixation are likely to be rather different, and provide information which helps maintain the spatial and linguistic context of words as a line of text is read. Moreover, because attentional resources are likely to be assigned most often in the direction of reading, these roles of leftward text may usually operate in a more passive (non-attentive) way, but be available for attentional processing when required (for example, when making a targeted regression; see also Jordan et al., 2016).

As a final comment, these findings were obtained for English text and the scene is now set for investigating how spatial frequencies either side of central vision affect reading in other languages, especially those read from right to left. For example, in languages such as Arabic, Urdu, and Hebrew (see e.g., Pollatsek et al., 1981; Jordan et al., 2014a; Paterson et al., 2014, 2015), progression through text requires identification of words to the left of fixation and regressions are made to the right. Thus, the asymmetrical influences of spatial frequencies observed in the present study may be essentially reversed for these languages. In addition, while it is well established that the left and right hemispheres differ in their processing of spatial frequencies and language (see Introduction), the precise role played by the two hemispheres in coordinating the processing of visual and linguistic information encountered to the left and right of fixation during reading remains to be fully determined. In particular, while upcoming text in languages read from left to right, such as English, will project initially to specialized processes of spatial frequency analysis and word recognition in the left hemisphere, upcoming text in languages read from right to left will project initially to the right hemisphere (see Jordan et al., 2011a). Differences in the functionality of the two hemispheres, therefore, may exert considerable influence on how text is processed dynamically in languages with different reading directions, and delineating these contributions is a crucial requirement for understanding fully the link between brain function and textual reading.

## Author Contributions

TJ: devised and designed the experiment and wrote the paper. VM: designed and analyzed the experiment and helped write the paper. SK: wrote the software, ran the experiment, and helped analyze the data. KP: devised and designed the experiment, helped analyze the data and helped write the paper.

## Conflict of Interest Statement

The authors declarethat the research was conducted in the absence of any commercial or financial relationships that could be construed as a potential conflict of interest. The reviewer SB and the handling Editor declared their shared affiliation, and the handling Editor states that the process nevertheless met the standards of a fair and objective review.

## Footnotes

1 The published literature contains no credible empirical demonstration of a precise split in hemispheric projections at the point of fixation in human vision (see Jordan and Paterson, 2009, 2010 for further explanation of this point).

2 As stimulus items were matched precisely across conditions, we report only analyses computed across participants (see Raaijmakers et al., 1999). However, for completeness, analyses were also computed across items and these *F*_2_ analyses showed the same patterns of effects.

3 It is worth noting that there was a tendency for L1-R13 windows to produce reading times that were actually shorter than normal for each spatial frequency, and this effect was especially noticeable for medium and high spatial frequency displays. This pattern suggests that, provided an area of normal text was present 13 characters to the right of fixation and 1 character to the left, a single band of spatial frequencies elsewhere in the display enabled readers to process text and propel the eyes forward more efficiently than normal, despite the impoverished visual nature of the information present outside the window. This pattern is also complemented by other measures of performance, including fixation counts and regressions. Conceivably, therefore, when reading normal textual displays, certain spatial frequencies present in a line of text may sometimes impair the reading process.
